# Sericin based nanoformulations: a comprehensive review on molecular mechanisms of interaction with organisms to biological applications

**DOI:** 10.1186/s12951-021-00774-y

**Published:** 2021-01-22

**Authors:** Gitishree Das, Han-Seung Shin, Estefânia V. Ramos Campos, Leonardo Fernandes Fraceto, Maria del Pilar Rodriguez-Torres, Kelli Cristina Freitas Mariano, Daniele Ribeiro de Araujo, Fabián Fernández-Luqueño, Renato Grillo, Jayanta Kumar Patra

**Affiliations:** 1 Research Institute of Biotechnology & Medical Converged Science, Dongguk University-Seoul, Goyangsi, 10326 Republic of Korea; 2 Department of Food Science & Biotechnology, Dongguk University-Seoul, Goyangsi, 10326 Republic of Korea; 3 Human and Natural Sciences Center, Federal University of ABC. Av. Dos Estados, 5001. Bl. A, T3, Lab. 503-3. Bangú, Santo André, SP Brazil; 4 Institute of Science and Technology of Sorocaba, São Paulo State University (UNESP), Av. Três de março, 511, Alto da Boa Vista, Sorocaba, São Paulo 18087-180 Brazil; 5 Departamento de Ingenieria Molecular de Materiales, Centro de Fisica Aplicada y Tecnologia Avanzada, Universidad Nacional Autonoma de Mexico, Blvd. Juriquilla 3001, 76230 Queretaro, Qro Mexico; 6 Sustainability of Natural Resources and Energy Programs, Cinvestav-Saltillo, 25900 Coahuila, C.P. Mexico; 7 Department of Physics and Chemistry, São Paulo State University (UNESP), Avenida Brasil, 56, Centro, Ilha Solteira, SP 15385-000 Brazil

**Keywords:** Sericin, Biomaterials, Drug delivery, Nanoformulation, Biomedical, Silk protein

## Abstract

**Background:**

The advances in products based on nanotechnology have directed extensive research on low-cost, biologically compatible, and easily degradable materials.

**Main body:**

Sericin (SER) is a protein mainly composed of glycine, serine, aspartic acid, and threonine amino acids removed from the silkworm cocoon (particularly *Bombyx mori* and other species). SER is a biocompatible material with economic viability, which can be easily functionalized due to its potential crosslink reactions. Also, SER has inherent biological properties, which makes possible its use as a component of pharmaceutical formulations with several biomedical applications, such as anti-tumor, antimicrobials, antioxidants and as scaffolds for tissue repair as well as participating in molecular mechanisms attributed to the regulation of transcription factors, reduction of inflammatory signaling molecules, stimulation of apoptosis, migration, and proliferation of mesenchymal cells.

**Conclusion:**

In this review, the recent innovations on SER-based nano-medicines (nanoparticles, micelles, films, hydrogels, and their hybrid systems) and their contributions for non-conventional therapies are discussed considering different molecular mechanisms for promoting their therapeutic applications.
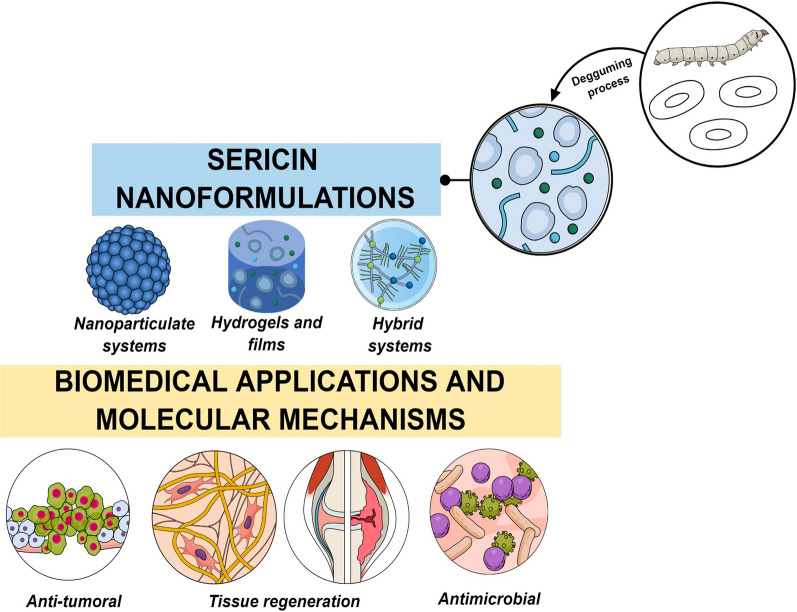

## Background

Silk is a fiber synthesized by some insects, for example, spiders, scorpions, bees among others[1], but its commercial use is limited due to extraction conditions, hence, the silk obtained from *Bombyx mori*, a domesticated silkworm species, is the most exploited [2, 3]. Silk is made up of two types of proteins: fibroin (65–85%) and sericin (SER, 15–35%). Fibroin is the core structure and SER is the gummy part that encloses fibers and holds them together [3]. Sericin is a water-soluble and adhesive protein, with a molecular mass between 20 and 400 kDa, produced by the silkworm’s gland (such as *Bombyx mori*, *Bombyx mandarins,* and other species) [4, 5]. It is a hydrophilic protein, constituted by hydroxyl, carboxyl, and polar amino acid groups such as glycine, alanine, arginine, leucine, aspartic acid, phenylalanine, isoleucine, valine, proline, glutamic acid, threonine, histidine, lysine, serine, tyrosine, methionine, tryptophan and cysteine [6, 7], which serine is the often amino acid. These polar chemical groups allow the formation of blends with other polymers by crosslinking, improving the mechanical resistance of SER-based biomaterials [4, 5]. SER is mainly described as SER-1, SER-2, and SER-3 types, determined by amino acids in their structure and molecular weight variation, being composed of a collection of polypeptides highly ample in the serine, aspartic acid (40%), and glycine (16%)[8]. It occurs in the incomplete unfolding state that encompasses a beta-sheet (35%) and random coil (63%) [9]. SER applications have been described in the food, cosmetics and biomedical fields. Concerning its biological activities, it was reported that it induces antioxidant, antityrosine, antiaging, antielastase, antibacterial, anti-inflammatory, antitumor and collagen production effects [10, 11].

Unlike fibroin, SER is not studied for long nor deeply as a component of nanomaterials. SER alone in its pure form presents high degradability, due to its extreme pH-induced instability, water solubility, and temperature [12]. All these features allow its association with polymers or other materials [13], in its bulk or nanosized form. Recently, silk-based nanomaterials have attracted attention due to the possibilities for obtaining biologically compatible and degradable materials with a range of biomedical applications as antimicrobials, antioxidants. Also, their low immunogenicity, high physicochemical stability, and long shelf life promote their scale-up production, being feasible as biomaterials [13, 14]. Fibroin and SER are the two commonly used derivative proteins from the silkworm used for the nanomaterial synthesis approved by FDA, in 2019 [15], expanding the possibilities for their biomedical use. Recent innovations and findings have reported pharmaceutical and cosmetics applications considering since modifications on isolation processes, for reducing its structural degradation, until functionalization strategies and drug-delivery systems design.

The SER structure maintenance is a challenge for developing SER-based materials and their formulations, because the conventional extraction processes (degumming) use conditions at high temperature and/or alkaline conditions, leading to protein chemical degradation, being avoided by the use of mild aqueous extraction [13]. Hence, chemical degradation control prevents changes in amino acid assembly (aggregation stands, β-sheets, and β-turns formation, etc.), favoring cell growth and attachment on SER membranes especially by existence of methionine and cysteine amino acids [15–17]. Also, low molecular weight SER peptides (< 20 kDa) have been used in hair care, skincare cosmetics formulations, health-related products, and also as formulation components, while the high molecular weight SER peptides (> 20 kDa) are utilized as the nanocarrier components, tissue scaffolds, functional biomembranes, etc. [18]. Besides, some reports highlight SER as the component of silk dressings containing antibacterial compounds and for natural polymer functionalization (gelatin, chitosan, alginate, collagen, and cellulose-derivatives) and as different forms (nanofibers, sponges, films, glues, nanoparticles, hydrogels, and 3D printed matrices [15, 19].

There is a variety of sericin-based nanomaterials usually as composites, for instance, films, nanoparticles used for as drug carriers taking advantage of its biological activities to improve them [20]. In this context, this review emphasizes the structural requirements for developing stable and effective SER-based formulations, as nanoparticulate systems, hydrogels, films, and association with different nanocarriers (hybrid systems), highlighting the recent innovations and findings on their cellular and molecular mechanisms for different biological applications such as anticancer, antimicrobial, antioxidant and wound healing processes.

### Meta-analysis update on SER-based studies

**Fig. 1:**
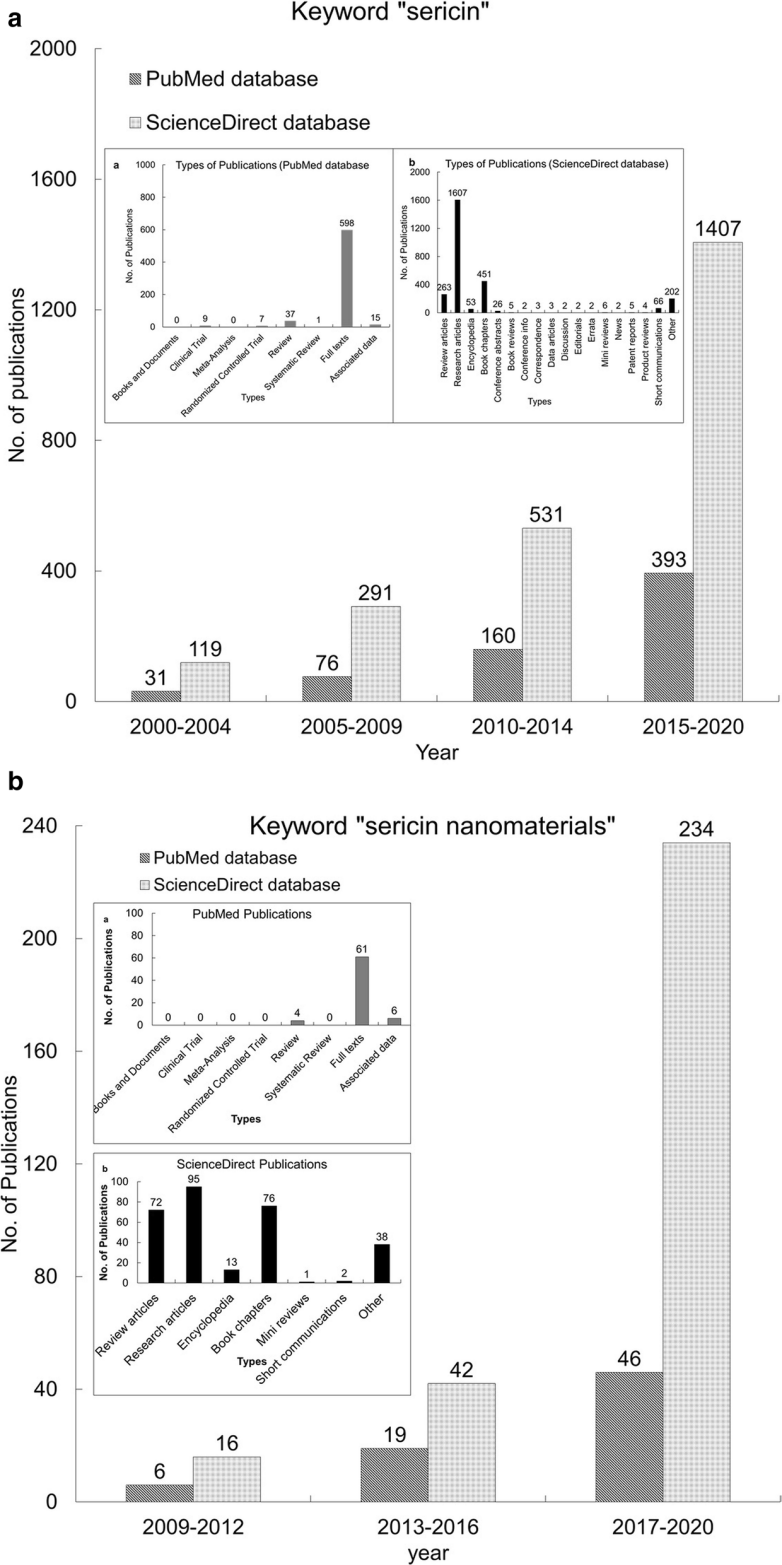
**a** Meta-analysis of publications on sericin till date as per the PubMed and ScienceDirect databases (Inset: **a** Types of publications as per the PubMed databases and **b** Types of publications as per the ScienceDirect databases); **b** A detailed meta-analysis on the publications related to SER nanomaterials in PubMed and ScienceDirect databases (Inset: **a** Types of publications as per the PubMed databases and **b** Types of publications as per the ScienceDirect databases)

Several studies on the SER protein dates to the year 1926. As per the PubMed database, around 700 articles have been published between 1926 and 2020 (https://pubmed.ncbi.nlm.nih.gov/?term=sericin). Among them, full texts account for 598 articles, whereas 37 review articles, 7 randomized controlled trials, 1 systematic review, and 15 associated data were published. It is stated that among the entire article published, 462 articles were during the last 10 years (2010–2020), out of which 314 articles were published during the last 5 years (2015–2020) and 81 articles are published in the last year (2019–2020) (Fig. 1a) (https://pubmed.ncbi.nlm.nih.gov/?term=sericin). Similarly, 2,704 articles have been reported to be published on SER in the ScienceDirect database since 1996–2020 and among them, 1607 are research articles, 263 are review articles (Fig. 1a) (https://www.sciencedirect.com/search?qs=sericin). Further, if we consider only the publications related to the biological properties of SER as well as its application in nanoformulations, only 71 articles were published from 2009 to 2020 as per the PubMed database, and among them, 4 were review articles, where very few details are presented on sericin (Fig. 1b) (https://pubmed.ncbi.nlm.nih.gov/?term=sericin+nanomaterials). Similarly, it is found that only 297 articles were published as per the ScienceDirect database from 2006 to 2020 (Fig. 1b) (https://www.sciencedirect.com/search?qs=sericin%20nanomaterials). Besides, it is reported in the literature that, nanomaterials related to SER comprise mainly research articles, book chapters, and reviews and but not clinical trials. This is not a rare outcome since SER has not been studied as part of nanoformulations, unlike fibroin, which is more studied. Trials on humans are still far from being carried out.

## Sericin extraction and purification processes

**Fig. 2:**
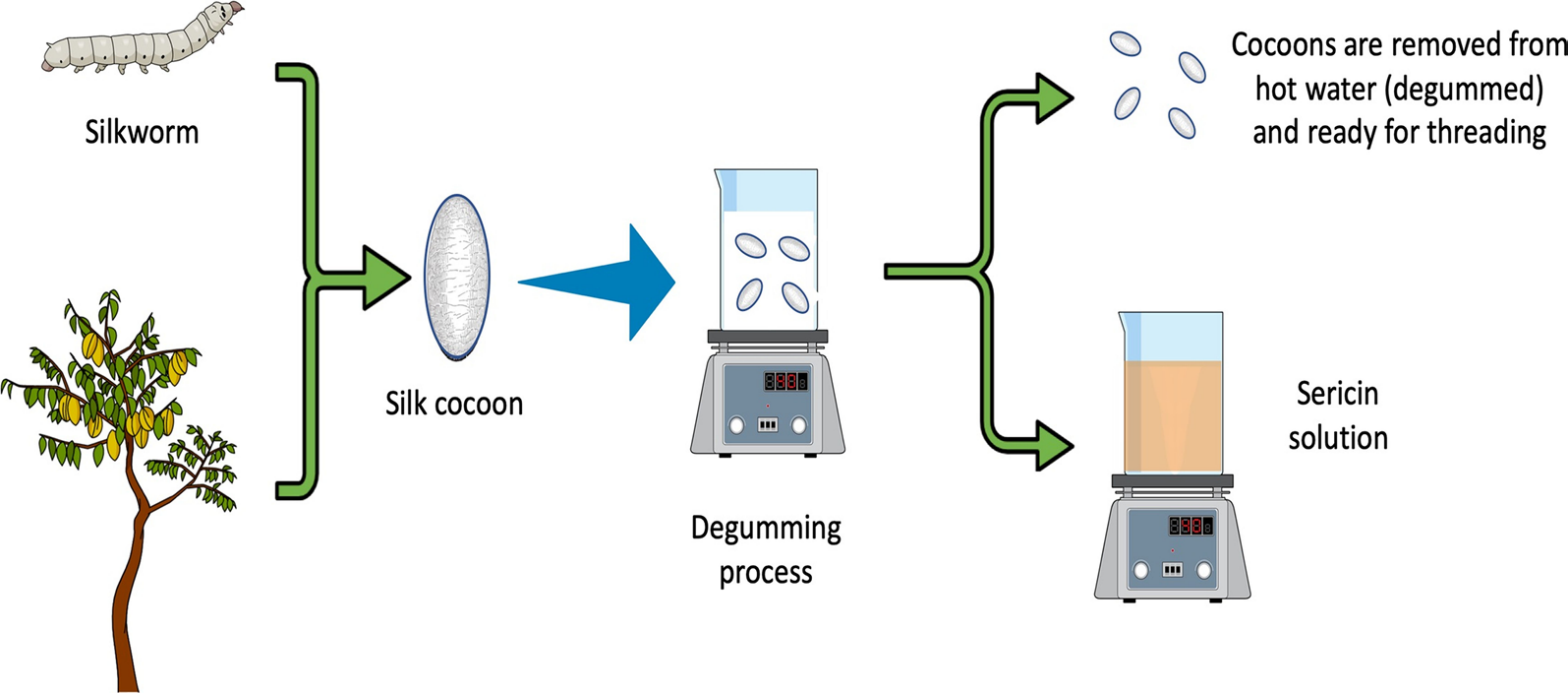
Sericin extraction procedure

The first stage in the manufacture of silk is the degumming process for SER extraction (Fig. 2), and it’s detachment from fibroin. Firstly considered as a residual product due to the silk final and commercial product is made up basically by fibroin [21]. The literature on variations for silk acquisition and more specific details about SER extraction can be consulted anywhere else based on aspects such as SER solubility in hot solutions, usage of soap, urea, enzymes as well as alkaline or acid substances or devices such as autoclaves, microwave ovens and the like, all of them oriented to avoid SER degradation [22]. In the second stage, it is necessary to isolate SER, which can be performed by filtration using membranes, ethanol precipitation, and freeze or spray drying [23, 24]. The choice of an extraction method is crucial because it determines properties like the molecular weight of the obtained SER.

### Extraction of SER

According to Kumar and Mandal [25], the conventional method to extract SER consists of boiling small pieces of cocoons (5 g L^−1^) in 0.02 M Na_2_CO_3_ for 30 min. By this process, fibroin is extracted and the volume is made to 10 mL followed by the separation of unwanted materials by the process of filtration and subsequent centrifugation. Further, the available solution is dialyzed using the dialysis membrane (cellulose tube with MWCO of 12 kDa), freeze-dried, and stored at − 20 °C until use [25]. Another method to extract SER was described by Aramwit et al. [23]. They used square pieces of fresh *Bombyx mori* cocoons and extracted them with pure water and autoclave at 121 °C and 15 psi of pressure for 1 h. Further, they collected the autoclaved sample, filtered it, and lyophilized the solution to obtain the SER powder. A second method for SER extraction is degradation by urea. This method was described by Tsubouchi et al. [26]. Fresh cocoon shells were mixed with 25 mL of 2 M urea in water and boiled at 100 °C for 5 min. Subsequently, they added 80 mL ethanol to the mixture, which resulted in the precipitation of the mixture, which was further centrifuged at 4000 rpm for 20 min and then solubilized in 3 mL of saturated aqueous lithium thiocyanate, and fractional precipitated with ethanol. Kurioka et al. [27], also described a method to extract acid and alkali-degraded SER using citric acid, tartaric acid, and succinic acid.

Recently, El-Fakharany et al. [28] described the process of separation and characterization of a novel silk-like protein. They extracted SER from bacteria able to produce a biopolymer called BNES, with a chemical composition similar to natural silk. The main advantage of this result is the possibility to produce SER by using large-scale bio-fermenters, reducing the scale-up costs, associated with the use of sustainable production sources (from bacteria). Currently, several ENMs have been used to separate different compounds or to catalyze chemical reactions. Therefore, it could be expected that nanotechnologies improve the extraction efficiency of SER using green chemistry or biological synthesis with a scare or null pollution and without toxic byproducts.

Chirila et al. [13], recorded four different extraction processes to find out the best process which could result in less hydrothermal deprivation of biomaterials from sericin. They used SDS-PAGE to examine the distribution by using molecular mass information followed by Fourier transform infrared-attenuated total reflectance (FTIR-ATR) spectrometry. Chirila et al. [13], concluded that the mild aqueous extraction procedure of a longer period than was carried out at a temperature of 50 °C was the best process to preserve the biomaterial properties among the four types of extraction procedures.

### Isolation and purification of SER

The isolation and characterization of SER components is an important task for determining the efficiency of sericin. Takasu et al. [29], isolated three main types of SER constituents (polypeptides having molecular masses of 400, 250, and 150 kDa) from the *Bombyx mori* cocoon and estimated them by SDS-PAGE. The characterization of SER fractions obtained from different extraction procedures was reported by Tengattini et al. [30]. Through several chromatographic methods and conditions, these authors found a close association between the average molecular weight of SER and the distribution of molecular weight and the hydrophilic/hydrophobic characteristics. Besides, they reported that the extraction procedure modifies the extracted components of SER. The extraction in the autoclave machine resulted in large proteoforms with a molecular weight (MW) of around 60 kDa while treatment with the bicarbonate resulted in a lower MW protein mixture (ten kDa).

Dash et al. [31], isolated sericin by using 8 M urea in 1% SDS and HSCH_2_CH_2_OH (2%) or 1% NaCl content. SER was cleaned by the gel filtration chromatography process. An isolated band of 200 kDa was identified by SDS-PAGE in both cases (reducing and non-reducing). Besides, they reported that the most often amino acids were glycine and serine. Wu et al. (2015) isolated and characterized a novel SER antifreeze peptide and determined its molecular mechanism related to the ice-binding property. They obtained a purified SER peptide called SM-AFP, which could be developed into beneficial cryoprotectants for frozen food processing. Besides, a broad list of options for the purification proteins is reported, such as by gel filtration chromatography, for example [31].

## Methods for preparation of SER based nanoformulations

Several methods are described for SER-based nano-carrier preparation, which are mostly based on enhancing of protein unfolding and decreasing of hydrophobic interactions between the chemical groups into the protein structure. To date, different simplified techniques such as desolvation, self-assembly, and salting-out are usually adopted for manufacture of silk-based nanoparticles, since these techniques uses mild processing conditions and easy to accomplish [32, 33]. Besides, other methodologies, such as capillary microdot printing, electro-spraying, microemulsion, supercritical fluid techniques, and electric filed applications are also used in the manufacture of silk mediated nanoparticles. For the SER-based nanoparticle synthesis, the most used techniques are desolvation, self-assembly, and crosslinking, which are discussed throughout the next topics [33, 34].

The desolvation is the most used technique to prepare protein-based nanoparticles since mild conditions are used. This method uses desolvating agent like acetone and ethanol to a water-based segment that contains the protein, which will result in the protein dehydration resulting in protein coil conformation. Some studies use the double desolvation method to get reduced and thin size nanoparticles [35]. Further, the protein amino groups can be cross-linked originating denser and more stable nanoparticles. The main disadvantages associated with this widely used technique is the addition of organic solvent as well as the utilization of toxic cross-linkers, such as glutaraldehyde, and low encapsulation efficiency [33, 34]. The desolvation method was used by Suktham et al*.* [36], to produce sericin nanoparticles stabilized with pluronic loading resveratrol. The nanoparticles loading resveratrol did not show toxicity for normal skin fibroblast (CRL-2522) while enhanced inhibition growth of colorectal adenocarcinoma cells (Caco-2) was observed. The nanoparticles internalization by Caco-2 cells were time-dependent, after 6 h less than 10% of nanoparticles were uptake by cells while after 24 h the internalization rate increased to 97%.

**Fig. 3:**
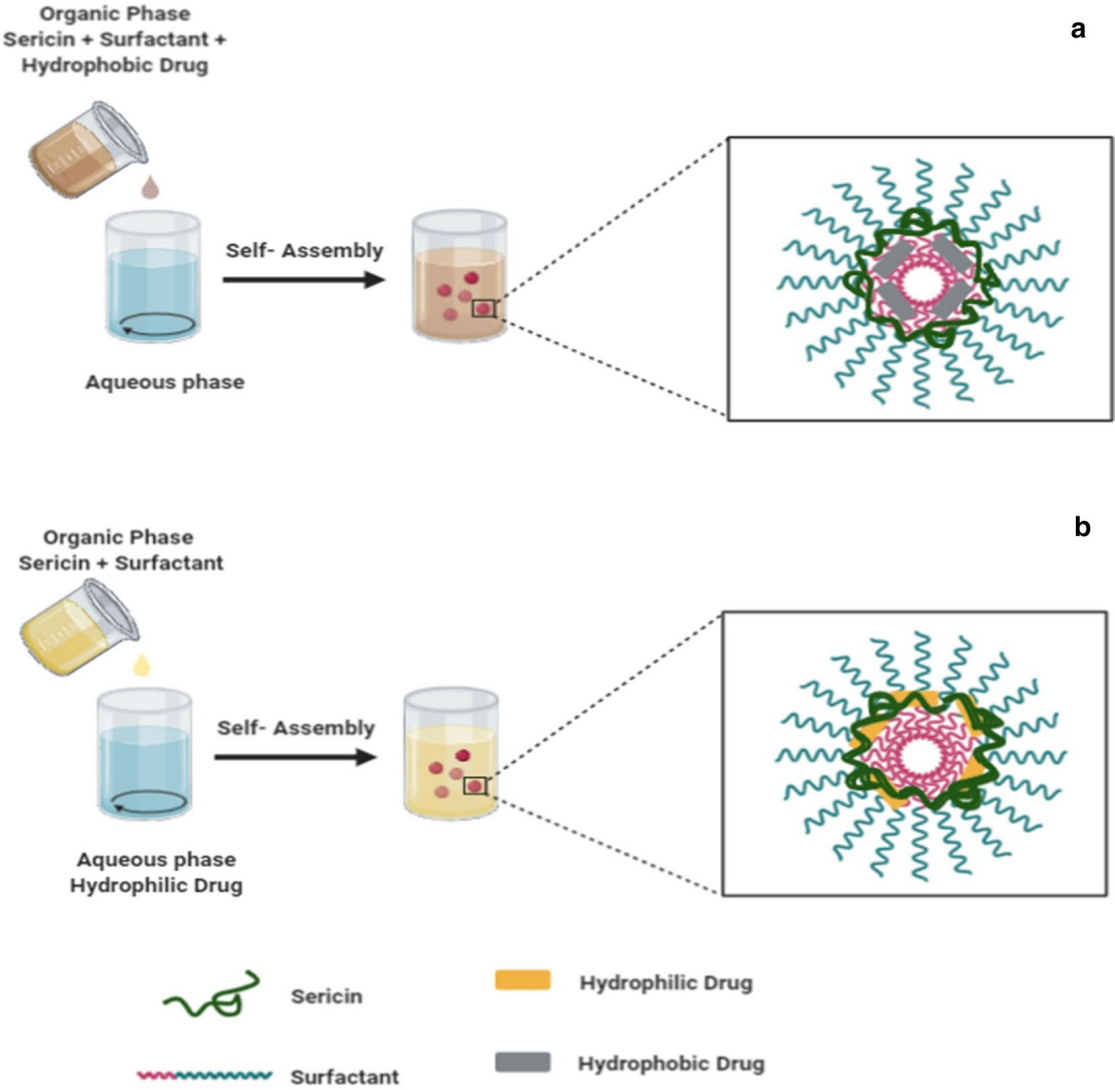
Schematic representation of self-assembly of sericin-based micellar nanoparticles. Incorporation of hydrophobic molecules in the hydrophobic core of sericin micellar nanoparticles (**a**) and encapsulation of hydrophilic drugs within the hydrophilic corona of sericin-based micellar nanoparticles (**b**). Created with BioRender.com

Spontaneous organization via self-assembly (Fig. 3) originating functional complexes is mediated by numerous weak non-covalent interactions of small building blocks [37]. Proteins when dissolved in an aqueous solution beyond the critical micelle concentration and critical solution temperature can form nano-sized masses [38]. This method is common in the utilization of other polymers to harden and stabilize the self-assembled micellar nanoparticles by crosslinking between the polymers chain [39]. The self-assembly technique was used by Mandal and Kundu [37], to synthesize sericin nanoparticles blended with Pluronic (F-127 and F-87) to load either hydrophobic drugs (paclitaxel) or hydrophilic (FITC-inulin) drugs. Also, nanoparticles loading paclitaxel showed greater cytotoxic effects to MCF-7 (breast cancer) cancerous cells related to the non-encapsulated drugs whereas they are non toxic to the normal cells. The mechanism behind the cell toxicity was related to cell apoptosis due to up-regulation of pro-apoptotic proteins (*Bcl*-*2*-associated X-*protei-*Bax), down-regulation of anti-apoptotic protein (B-cell lymphoma *2 -*Bcl-2) and the regulatory protein PARP (Poly (ADP-ribose) polymerase) degradation.

**Fig. 4:**
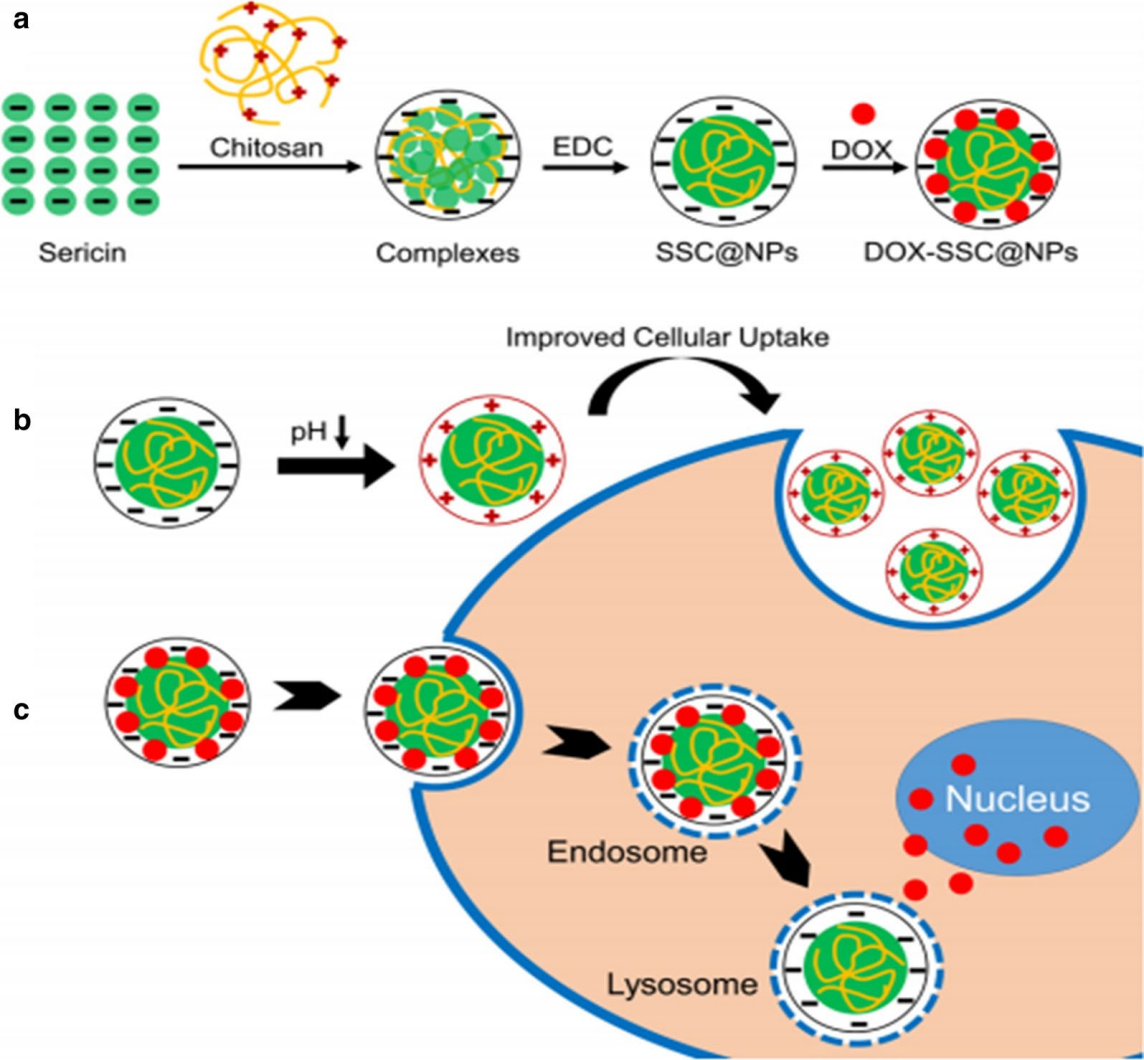
a Schematic Representation of Formation of SSC@NPs and DOX-SSC@NPs, **b** Decrease in pH Inducing Surface Charge Reversal of SSC@NPs, Facilitating Cellular Uptake of SSC@NPs, and **c** Intracellular Drug Release and Distribution of DOX-SSC@NPs. Reprinted with permission from Hu et al. [40]

Another simple technique to synthesize protein-based nanoparticles is cross-linking methods (Fig. 4). Several types of crosslinkers are used to synthesize protein-based nanoparticles, such as chemical, ionic, enzymatic, and thermal. The most used crosslinker agent is glutaraldehyde, which promotes multifunctional crosslinking into protein networks by linking free amino groups [39]. However, due to toxic concerns, the utilization of natural crosslinker has been extensively investigated [33, 34]. Hu et al. [40], produced charge-reversal SER-based nanoparticles by using a two-step cross-linking method that utilizes a physical reaction between the sericin and chitosan followed by a chemical EDC crosslinking aiming to increase the cellular uptake of nanoparticles loading anticancer drug doxorubicin. Nanoparticles showed pH-responsive charge-reversal characteristics, for example, it becomes negatively charged in the neutral pH and positively charged in the acid condition (pH 6), which subsequently forms a characteristic feature for differentiating the tumors and its surroundings. The cellular uptake was sixfold higher in HeLa cell incubated at pH 6 in comparison to those incubated at pH 7.4 and the uptake nanoparticles were accumulated into the lysosomes and the drug released to the nucleus of cancerous cells.

## Potential biological applications of SER

Different approaches have been studied for SER, especially related to future large-scale production, considering their various biological applications such as antimicrobial, UV-protectant, anti-aging, antioxidant, hepato-protective, anti-inflammatory, anti-cancer, anti-viral, wound healing, among others. Besides, SER has potential use in association with engineered nanoparticles (ENPs) for cutting-edge therapeutic applications.

Tahir et al. [41], evaluated the antibacterial action of SER-conjugated silver ENPs (SCS-ENPs). The biogenic ENPs reduced the growth of *Escherichia coli*, *Staphylococcus aureus*, and *Klebsiella pneumoniae* greatly. Besides, they showed that SCS-ENPs were stable at different temperature and pH and effective against bacteria. Therefore, they concluded that SCS-ENPs possess a pronounced antibacterial activity, suggesting their far-reaching applications as a low-priced and established antimicrobial agent. Lv et al. [42], synthesized Ag-ENPs conjugated to sericin (AgENPs-Sericin) with the potential to destroy *E. coli* and *S. aureus*. Therefore, this conjugated nanoparticle could serve as a promising antimicrobial agent against the sexually transmitted infections (STDs) [42].

Pankongadisak et al. [43], prepared gentamicin sulfate (GS)-conjugated Poly(L-lactic acid)- SER hybrid scaffolds (GS-PLLA-SHS). These engineered nanomaterials (ENMs) exhibited promising potential against the *S. aureus* TISTR 1466and *E. coli* TISTR 780. Besides, GS-PLLA-SHS were non-toxic to the MC3T3-E1 cells, endorsing their use as a potential candidate for bone tissue engineering applications. One year before, chitosan, sericin, and glycerophosphate conjugated with the longan seed extracts were also used for the above said purpose [44]. Veiga et al. [45], discussed different manuscripts on recent advances for sericin/Ca_3_(PO_4_)_2_ composites production, with application in bioengineering, pharmaceutic, cosmetic, food, and environmental fields.

Other biomedical applications such as artificial skin, wound dressing, and tissue engineering was described for the dialdehyde carboxymethyl cellulose and silk sericin [46, 47]. Nagai et al. [48], also demonstrated the therapeutic potential of SER for epithelial corneal regeneration in mice using the mixture of SER and solid Mg(OH)_2_ ENMs. Shah et al. [49], developed chitosan-SER-silver nanocomposite (CSSN) films. Their findings strongly suggested the use of CSSN for wound care applications. Clinical trials have been done using sericin composites as wound dressings (NCT01539980, NCT02643680, NCT02091076, NCT04299126).

The inhibitory potential of SER against the UV-influenced melanogenesis was reported by Kumar and Mandal [50] which was investigated by the tyrosinase activity, intracellular melanin content, and the levels of ROS in the mouse melanoma. They found that SER significantly decreased the ROS production and the cellular melanin content in UV irradiated melanocyte cells compared to the SER control cells. Also, they reported the preparation of a skincare formulation by adding SER from *Antheraea assamensis*. The formulation flow properties of the prepared skincare formulation was found out not being affected by incorporation of SER. Consequently, SER is an antioxidant molecule with potential use in skincare cosmetics.

**Fig. 5:**
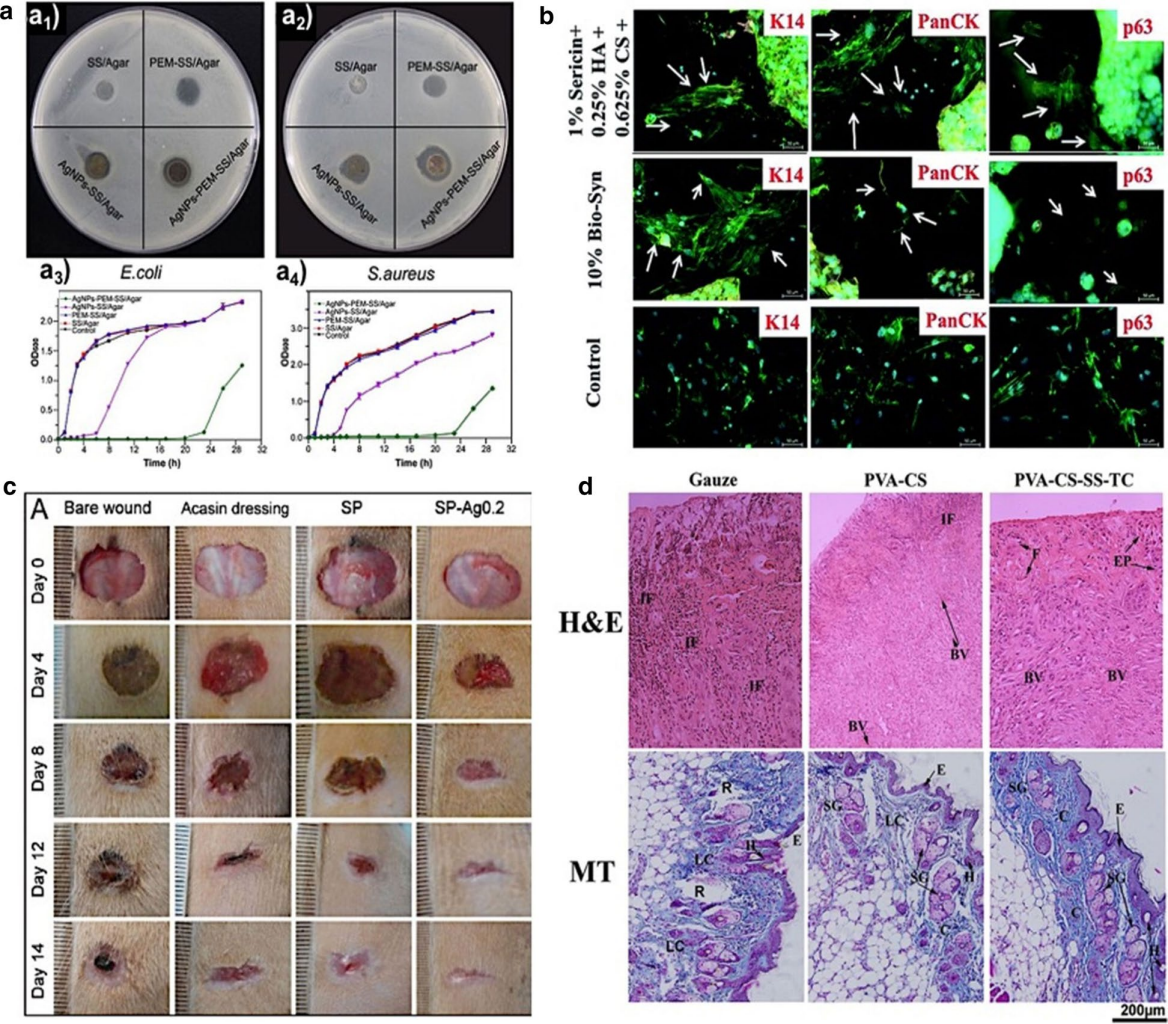
SER nanoformulations as antimicrobial and regenerative of tissues; **a** Antimicrobial effect of AgNPs-SER films against *E. coli* (**a**_**1**_) and *S. aureus* (**a**_**2**_) and growth curves of the bacteria; *E. coli* (**a**_**3**_) and *S. aureus* (**a**_**4**_). Reproduced under the terms and conditions of the Creative Commons Attribution (CC BY) license (http://creativecommons.org/licenses/by/4.0/) from Liu et al. [63] (originally Fig. 7); **b** SER scaffolds were immunostained using Keratin 14 (K14), Np63α (p63), and Pan-cytokeratin (PanCK) monoclonal antibody. Reproduced under a Creative Commons Attribution-Non Commercial 3.0 Unported Licence from Bhowmick et al. [67] (originally Fig. 4); **c** Photo of the wound healing effect of the SER nanoformulation used as dressing in vivo mouse (Reproduced with permission from Tao et al. [68] (originally Fig. 11A), and **d** images of hematoxylin–eosin (H&E) and Masson's trichrome (MT) staining of wound sites 7 days after treatment. Reproduced with permission from Bakhsheshi-Rad et al. [69] (originally Fig. 8))

SER hydrogel is a dressing material with outstanding properties and antimicrobial activity. Tao et al. [51], harnessed these properties to develop a novel and non-toxic hydrogel with antimicrobial activity and super-absorbent characteristics with potential use as a wound dressing. In a fast and easy process, they blended silk SER with poly (vinyl alcohol) (PVA) to synthesize a SER/PVA hydrogel by successive freeze-thawing processes. Besides, SER/PVA hydrogels were able to load and release small molecules and Ag-ENPs with excellent biocompatibility. A film with long-lasting antibacterial ability was synthesized by Liu et al. [52]. It was made with Ag-ENPs-polydopamine-sericin/Agar and showed excellent cytocompatibility on the fibroblast NIH/3T3 cells and great potential as a novel wound dressing. Gilotra et al. [53], also studied the potential of silk sericin based nanofibrous mats for wound healing applications using PVA-SER blended mats. According to them, SER-based dressing is a potential ENMs candidate for the treatment of chronic wounds like diabetic foot ulcers. The preparation and evaluation of levocetirizine-loaded emulgel containing tamanu oil and SER for atopic dermatitis treatment were carried out by Pal et al. [54]. They enhanced the therapeutic potential of emulgel in terms of reduced scratching frequency and erythema score, as witnessed by in vivo pharmacodynamic studies. Then, SER-based emulgel could be an alternative appropriate dosage form for the treatment of atopic dermatitis. Even though SER has potential applications in the biological and engineering sector, it is an environmental concern because it is an unutilized byproduct of the textile industry that leads to environmental contamination due to the high oxygen demand for its degradation by microbes [11]. Antibacterial materials have gained prominence in the last years due to its use in medical devices and implants [55]. Although the use of silver nanoparticles is already well known in this area, current studies show that the association of sericin with silver nanoparticles (AgNPs) may increase the antibacterial activity of these materials, since SER has antimicrobial and antioxidant activity [10, 56]. Moreover, SER has been used as a dispersant and stabilizing agent of some nanoparticles synthesis [57]. The antibacterial activity of SER in bionanomaterials can be found in several compounds, such as AgNPs/Sericin/Agar Film [58], poly(ethylene terephthalate) fibers with sericin-capped AgNPs [59], poly-L-lysine-coated sericin nanoparticles [60], Sericin-NIPAAm-AgNPs Hydrogel, sericin/PVA blend film with AgNPs [61], Sericin/Glycerol Films Coated with AgNPs [62], among others. In particular, Liu et al. [63], developed AgNPs and polyelectrolyte membrane (PEM) modified sericin/Agar films to control *E. coli* and *S. aureus* as observed in Fig. 5a. Furthermore, other nanoformulations containing SER have been used with antibacterial activity for potential use in medical applications, such as sericin-capped gold nanoparticles [64], ZnO nanoparticles on Sericin/Polyvinyl Alcohol Composite Film [65], etc.

SER nanoformulations can also be found for regenerative tissues [66], through biomaterials for wound healing [53] and artificial skin [67]. Studies have shown that SER can increase skin keratinocytes and fibroblasts proliferation [10], as well as may promote wound healing since it may facilitate collagen deposition [26]. For instance, Bhowmick et al. [67], developed a skin substitute for 2nd burn care composed of mPEG–PCL–grafted-gelatin (Bio-Syn)/hyaluronan/chondroitin sulfate/SER nanofibers, and it was observed that the nanofibers (mainly containing 1% of sericin) exhibited enhanced levels of epithelial protein expression (Fig. 5b) as well as, better performance as burn wound in rats model compared to the commercial products [67]. Similar wound dressing devices are also described by Tao et al. [68] and Bakhsheshi-Rad et al. [69], when fabricated silk sericin-based sponges with AgNPs and sericin-based poly(vinyl alcohol)/chitosan/tetracycline porous nanofibers, respectively. Such materials showed enhanced wound healing properties when evaluated in animals, and exhibited a better re-epithelialization, and collagen deposition compared with the control (Fig. 5c, d). Besides that, other sericin regenerative devices can also be found for regenerating articular cartilage [70], sheath for peripheral nerve regeneration [71], cardiac patches to be used to repair heart after myocardial infarction [72], and bone tissue engineering [73, 74].

## SER nanoformulations: biomedical and pharmacological properties

Nano-based formulations have been proposed as tools, which hold the promise to modernize the delivery systems field. Protein-based nanocarriers have been widely studied for nanoformulation production, due to their inherent properties, such as biodegradability, biocompatibility, self-organization, and low toxicity [10, 33]. Furthermore, due to the amphiphilic properties of proteins is it possible to carry both hydrophobic and hydrophilic compounds. Besides, proteins are easily functionalized because of the large quantity of hydroxyl, amino, and carboxyl groups, which are subject to chemical modification. Amongst the proteins, silk proteins, have been used to develop nano-based formulations owing to its physicochemical and biomedical properties [34].

The exceptional biological characteristics of SER, such as anti-inflammatory, anticoagulant, antioxidant, antimicrobial, and anticancer activity, make SER-based formulations as promising delivery systems and tissue scaffolds [19, 75]. SER has been described as a protein with extensive uses in the medical field with a promising future in tissue engineering, diagnosis, and disease therapies [32]. This protein biopolymer has been used as inert or non-inert supports to fabricate films, sponges, fibers, patches, scaffolds, hydrogels, micro, and nanostructures [75, 76]. Among these materials, nanostructures have been highlighted considering their antibacterial effects, wound healing, artificial skin, articular cartilage, scaffolds for tissue regeneration, and small molecules delivery systems [77]. Table 1 summarizes the nanoformulations based on SER and their applications.

**Table 1:** SER-based nano-formulations for biological applications

Nanomaterial	Active compound	Method of synthesis	NPs Properties	Main characteristics/advantages	References
Sericin-chitosan	Doxorubicin	Cross-linking method	MD: 231 ± 7.3 nm; ZP: − 6.25 ± 1.2; PDI: 0.067	pH-dependent release of doxorubicin and increased cellular uptake in tumor acid environment. Self-stabilization and increased colloidal stability after freeze-drying and resistance of plasma protein adsorption. In vivo assays showed significant anti-tumoral activity with reduced doxorubicin cardiotoxicity	[40]
Sericin- poly(ethylene glycol) (PEG) nanoparticles	-	Self-assembly	MD: 197.3 ± 46 nm	Spherical nanoparticles are formed by hydrophobic interaction between sericin and PEG	[78]
Sericin nanoparticles coated with poly-L-lysine	Plasmid encoding a green fluorescent protein	Desolvation method	MD: 244.00 ± 8.48 nm; ZP: -29.7 ± 2.54 MV; PDI: 0.35 ± 0.07	Plasmid DNA was strongly packed to the NPs surface, which leads to significant transfection of mouse fibroblast after 72 h. No toxicity was observed	[79]
Sericin-Poly(ethylcyanoacrylate) Nanospheres	Fenofibrate	Interfacial polymerization	MD: 175 ± 12 nm; ZP: − 32.8 ± 1.0 mV; PDI: 0.201	Increased ~ twofold the drug absorption in the gastrointestinal tract compared to a non-encapsulated drug, reducing the levels of total cholesterol (TC), triacylglycerols (TG), very low-density lipoproteins (VLDL), and low-density lipoproteins (LDL)	[80]
Sericin-genipin nanoparticles	Atorvastatin	Desolvation-genipin crosslinking method	DM: 166 ± 0.30 nm; ZP: − 38.28 mV; PDI: 0.225	Nanoparticles (10 mg.kg^−1^) enhanced the antihyperlipidemic activity in mouse models in comparison to a non-encapsulated drug at the same concentration. No cytotoxicity was observed to murine (J774) cells	[81]
Sericin-folate nanoparticles	Doxorrubicin (DOX)	Self-assembly and chemical crosslinking	DM: 53.8 ± 17.6 nm; ZP: -15.15 ± 1.51 mV	The release of DOX is ~ fivefold higher in an acid environment than in a neutral condition. NPs were target to folate receptors of cancer cells and displayed good hemo-compatibility	[7]
Sericin-chitosan nanoparticles	Doxorrubicin (DOX)	Physical and chemical crosslinking	DM: 231 ± 7.3 nm; ZP: − 6.25 ± 1.2 mV; PDI: 0.067	pH-dependent release of DOX and increased cellular uptake in tumor acid environment (pH 6)	[82]
Sericin-silver nanoparticles	-	Green reduction using sericin as reductant	DM: 4 – 20 nm	Significant inhibition of *S. aureus* growth at 25 mg.L^−1^ of nanoparticles. No cytotoxicity was observed to murine cells (3T3) under this concentration	[57]
Sericin nanoparticles	Resveratrol	Desolvation method	DM: 183.43 ± 7.23 nm; ZP: ~ -20 mV; PDI: 0.2	Significant inhibition of cancerous cell growth (human colorectal adenocarcinoma, Caco2) while no cytotoxicity was observed to normal skin fibroblasts (CRL-2522)	[18]
Sericin-gellan gum-rice bran albumin nanocomposites	Doxorubicin	Self-assembly	DM: 218 nm; ZP: -7.43 mV	IC_50_ of DOX for breast cancer cell line (MCF-7) was reduced 1.8-fold after encapsulation. The survival rate of cancer cells was reduced by 42% after treatment with nanocomposites	[83]
Sericin poly(γ-benzyl-L-glutamate) (PBLG) micelles	Doxorubicin	Self-assembly	DM: 111.1 ± 0.7 nm; ZP: -25.4 ± 0.76 mV; PDI: 0.17 ± 0.01	The drug release was accelerated in the lysosomes under acid pH. Micelles induced 1.5-fold higher apoptosis in the breast cancer cell line (MCF-7) concerning the non-encapsulated drug. Suppression of tumor growth in vivo was 70% higher in mice-model treated with micelles in comparison to control	[84]
Sericin-cholesterol-folate nano micelles	IR780	Self-assembly	DM: 140 nm	Cancerous cells (Human papillomavirus-related endocervical adenocarcinoma, BGC-823) containing folate receptors efficiently internalized the nanoparticles, which results in cell toxicity after laser irradiation at 808 nm	[85]
Sericin nanoparticles	Curcumin	Desolvation method	DM: 278.15 ± 53 nm; ZP: -23.0 ± 3.59 mV; PDI: 0.54 ± 0.09	The best nanoparticle physicochemical characteristic was achieved with 1 mg.mL^−1^ of sericin, which results in 84.7% of encapsulation entrapment	[86]
Sericin nanoparticles coated or not with poly-L-Lysine	-	Desolvation method	DM: 16 to 156 nm; ZP: -34 to + 36.2 mV; PDI: 0.8 to 1	NPs produced with sericin (30–50 kDa) positively charged showed the strongest antibacterial effects in *S. aureus* and *E. coli* strains by enhancing ROS production	[60]
Sponge nanocomposite of sericin-silver nanoparticles	-	Green reduction using sericin as reductant	DM: 111.1 to 175 nm	Both nanoparticles and sponge nanocomposites induced antibacterial activity against *S. aureus* and *E. coli.* Antibacterial activity of sponge nanocomposites was concentration-dependent, being the best results achieved at 100 ppm of AgNPs	[87]

*MD* mean diameter, *ZP* Zeta potential, *PDI* polydispersity index

**Fig. 6:**
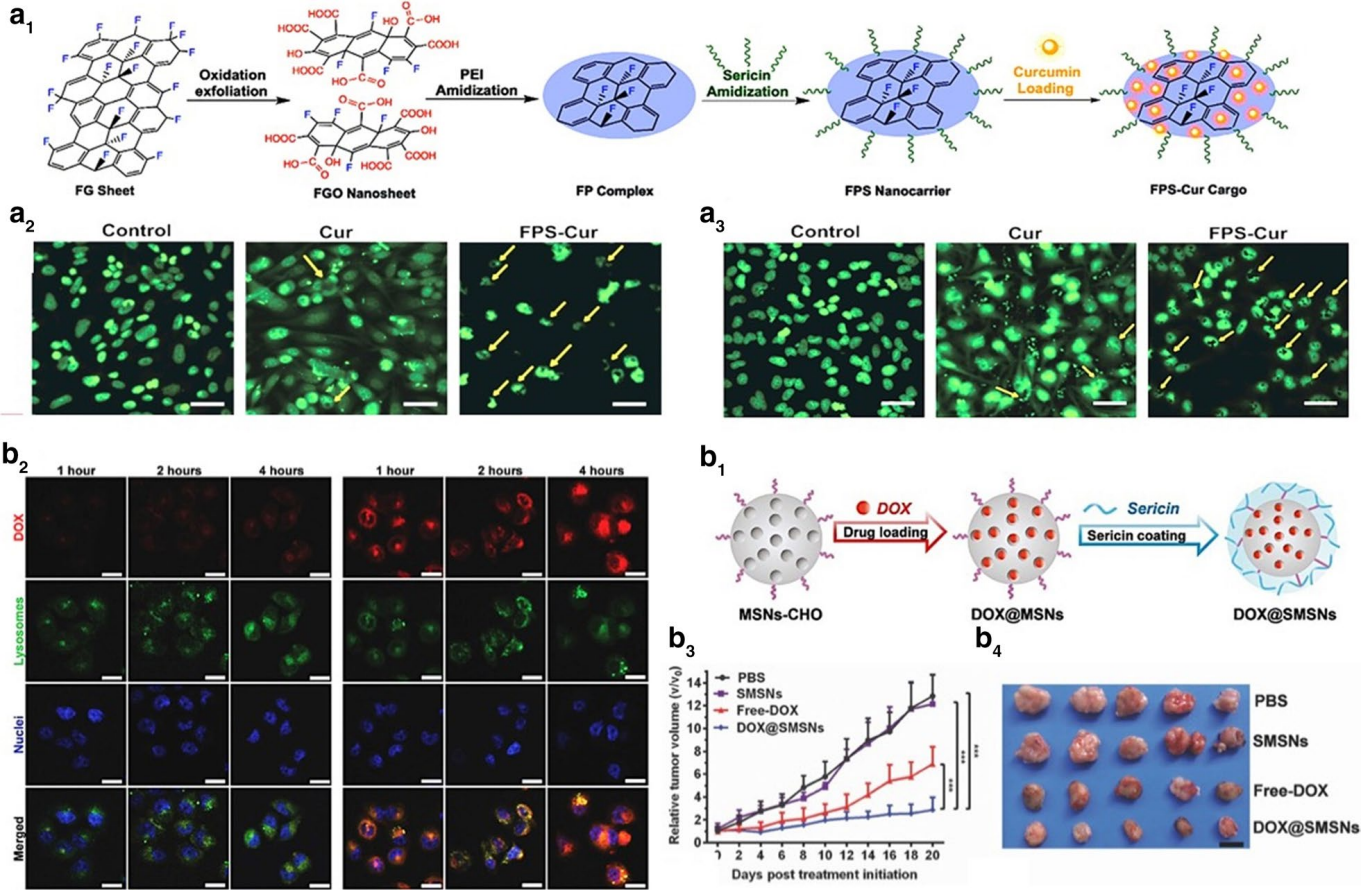
Examples of SER drug delivery nanoformulations for cancer treatment. **a**_**1**_ Schematic representation of the construction of the pH-sensitive charge-reversal system based on curcumin (Cur) loaded fluorinated graphene oxide (FGO) modified with polyethyleneimine anchored to sericin-polypeptide (FPS); **a**_**2**_ Apoptotic morphology study using cell nuclei stained by DAPI dye in PC-3 (**a**_**2**_) and HeLa cells (**a**_**3**_) treated with PBS (control), free curcumin (Cur), FPS-Cur for 6 h treatment. Reproduced with permission from Jahanshahi et al*.* [89]; **b**_**1**_ Schematic illustration of the synthesis procedure of doxorubicin (DOX) loaded sericin-coated mesoporous silica nanoparticles (SMSNs); **b**_**2**_ Cellular uptake of free DOX (left) and DOX-loaded SMSNs (right) for 1, 2 and 4 h; **b**_**3**_ The relative tumor volume of MCF-7/ADR tumor-bearing mice over 21 days; **b**_**4**_ Representative tumors isolated from the mice after the treatment. Reproduced with permission from Liu et al. [90]

Among the studies reporting the development of SER-based nanocarriers, the preparation of stable and effective systems is the main concern. Considering the easy functionalization of SER molecule, strategies for producing more specific and environmental-sensitive systems are used especially for tumor targeting. Several production techniques have been described to prepare these types of nanoparticles [75, 77], and beyond that, some nanocarriers have smart release capacity, which means that they can release drugs in specific conditions, for example when changing the pH values [7, 40, 83, 84, 88]. In particular, Jahanshahi et al. [89], developed a sericin anchored fluorinated graphene oxide (FGO) pH-responsive carrier for a controlled release of curcumin (Fig. 6a1). Such nanocarrier presented high load carrying capacity, improvement of nuclear-targeted delivery of cells, and enhanced curcumin cell internalization, as well as promoted apoptosis in SkBr3 (human breast/mammary cancer cells), HeLa (cervical cancer cells), and PC-3 (prostate cancer cells) cancer cells (Fig. 6a2, a3) (purchased from ATCC (American type culture collection) and Sigma-Aldrich) [89]. Furthermore, Liu et al. [90], fabricated doxorubicin (DOX) loaded sericin-coated mesoporous silica nanoparticles aiming a pH/protease dually responsive drug delivery in the acidic environment of the lysosome (Fig. 6b1). Such nanoparticles showed a better cellular uptake in comparison with DOX non-encapsulated as a function of time (Fig. 6b2), and significantly reduce the growth of DOX-resistant to the breast cancer cells (Fig. 6, b3-4) [90]. Positive outcome are also shown by DOX loaded sericin/poly(γ-benzyl-L-glutamate) nanomicelles [84], sericin/chitosan-based nanoparticles [91], and folate-conjugated sericin nanoparticles [7].

**Fig. 7:**
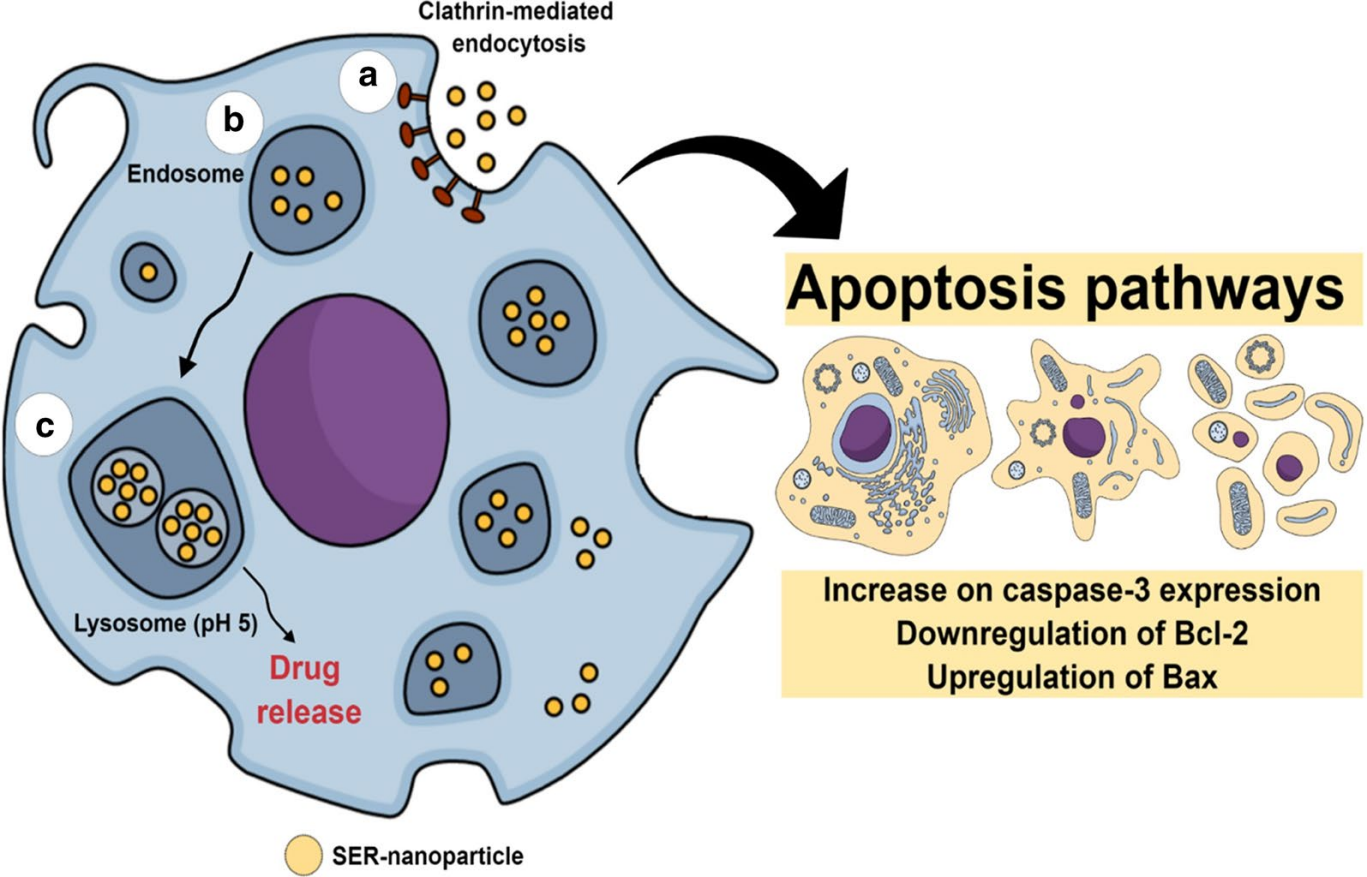
Representative molecular mechanism of SER-based nanoparticles for cancer therapy. **a** clathrin-mediated SER-nanoparticles internalization; **b** nanoparticles endocytosis; **c** nanoparticles uptake by lysosomes and drug release into acid pH. Sequential cellular events are apoptosis pathways activation. *SER* sericin

Despite, DOX is one of the most used anticancer agents, other drugs were also tested as guest molecules on SER-nanoparticles. Resveratrol-loaded SER-Pluronic F-68 nanoparticles evoked more pronounced cytotoxic effects for colon tumor cells concerning skin fibroblasts, resulting from nanoparticle accumulation into cancer cells in association with the enhanced permeation and retention (EPR) effect. Other active ingredients such as vitamin B12-conjugated sericin micelles [92], are being associated with sericin nanoformulations. Also, Mandal and Kundu [37], reported the cytotoxic effects evoked by micellar nanoparticles composed of paclitaxel-loaded SER-Pluronic F-127, showing fast internalization by breast cancer cells followed by Bax upregulation and Bcl-2 downregulation, both pro-apoptotic and anti-apoptotic proteins, respectively. In general, for cancer chemotherapy, SER-based nanoparticles exhibited promising effects attributed to molecular mechanisms involving integrated events initiated by (i) drug-loaded SER-nanoparticles internalization due to clathrin-modulated endocytosis; (ii) nanoparticles disintegration followed by drug release into lysosomes acid pH (observed into tumor environment); (iii) cell death pathways induction such as apoptosis mediated by caspase-3, downregulation of Bcl-2 and upregulation of the Bax proteins; and (iv) nanoparticles accumulation in tumor cells, as a result from EPR-effect [19, 37]. Isolated SER anti-tumoral effects were previously reported considering its integrated pro-apoptotic activities like decrease in caspase-3 expression, downregulation of Bcl-2, and human colorectal cancer cell cycle arrest [93]. All those features highlight the benefits of using SER-based nanocarriers for cancer therapy. Another system designed for doxorubicin release was described by Hu et al. [40], using SER as nanoparticles dispersion stabilizer and cryoprotectant, due to its ability for reducing the adsorption of plasma proteins. The nanoparticles' physico-chemical stability and the decrease of drug side effects were important advantages described for the systems since DOX administration is frequently associated with cardiotoxicity, due to its affinity by cardiolipin. The in vivo antitumor efficiency assays revealed that nanoparticles evoked pronounced tumor cell necrosis were biocompatibility (attributed to hydrophilicity and the negative surface of SER nanoparticles) and were sufficiently self-stabilized to be administered by intravenous route. Additionally, in vitro tests showed no effects on coagulation cascade neither hemolysis. Figure 7 shows a representative mechanism of SER-based nanoformulations for cancer therapy.

Folate-SER-based nanoconjugates were designed for loading IR780, a near-infrared hydrophobic and photosensitive dye. By achieving high entrapment percentage (88%) and controlled size (140 nm), micelles antitumor effects were increased after folate-positive gastric cancer cells irradiation, highlighting the system's application for cancer phototherapy [85]. A similar strategy was presented by Guo et al. [92], by developing vitamin B12-conjugated SER-micelles, to potentialize their removal by tumor gastric cells that overexpress CD320 receptors. Internalized micelles activated the caspase9/caspase3 apoptosis pathway reversing the drug resistance phenotype. Similarly, Huang et al. [7] reported the synthesis of multifunctional SER-nanoparticles was based on two successful steps compressing the conjugation of doxorubicin with SER, due to covalent hydrazine bonds capable to respond intracellular pH variations, and the incorporation of a folate molecule covalently grafted to the doxorubicin-SER conjugate. Their efforts lead to the synthesis of a nanoparticle system with differential properties: (a) self-assembly capability attributed to the formation of the DOX-SER amphiphilic conjugate; (b) recognition by folate receptors on tumor cells; (c) pH-triggered doxorubicin release into lysosomes acidic environmental and; (d) small size (~ 54 nm) and negatively charged surface (-15 mV), reducing the uptake by the bloodstream. Taken together, all those features allowed the folate positive human oral epithelium carcinoma cells to uptake the nanoparticles by endocytosis, and doxorubicin was delivered into lysosomes, demonstrating the nanoparticles' potential effectivity for delivering chemotherapeutic drugs. Further examples of sericin drug delivery can be found in Elahi et al. [19] and this is only the beginning of the countless pharmacological benefits that this protein will bring to the development of medicine.

Besides its use as a potential drug-delivery carrier, SER has been used for synthesizing scaffolds used in tissue engineering for epithelial and connective tissue repair [45, 94]. Microbial contamination in wounds could result in severe sepsis, due to wound healing and antimicrobial properties of sericin, studies were performed to develop efficient wound healing formulations based on sericin [95, 96]. Vulpe et al. [97], obtained scaffolds composed of collagen, hyaluronan, and SER, with adequate morphology and physical–chemical features for applications as controlled drug-delivery systems. These scaffolds showed porous structure, strength, and stability for their application as skin tissue repair. Also, they can control the drug release profiles in a simulated biological medium. A similar finding was reported by Hu et al. [82], when they studied the self-stabilized SER-based NPs, which could be a secure and effective drug carrier for intravenous administration. Lamboni et al. [17], also reported that SER is a versatile material for tissue regeneration and drug-delivery applications, considering its physicochemical features and the nature of its origin. Also, by demonstrating the influence of SER-molecular size and the nanoparticles surface charge, variation is possible to predict some biological effects, such as pronounced antibacterial (bacterial membrane damage due to blebbing formation) and antioxidant activities for positive-charged nanoparticles composed of SER with molecular weight interval from 30 to 50 kDa [86].

Due to their low-toxicity, biodegradability, and biocompatibility, sericin-based nanoformulations have been studied as a potential carrier for the delivery of both small drugs and bio-macromolecules, increasing their absorption and bioavailability [10]. Furthermore, due to the aqueous solubility of sericin, it is possible for nanoparticle synthesis under mild conditions [19]. However, nanoparticles produced only with sericin show poor stability, which is influenced by pH changes, temperature, and water solubility [98]. To overcome this, the addition of stabilizers, crosslinking agents, and mix with other polymers (natural or synthetic) is highly recommended to avoid burst release and drug leakage due to poor stability [75]. Although in the last years the sericin-based formulations have been capturing the attention of the scientific community, these nano-based formulations are far from reaching the clinics [75]. As discussed in this section, very good results were reported for SER-based nanoformulations, especially as anticancer therapies. There is an interesting study on the sericin-specific immune responses. An in vivo study by Jiao et al. [99], suggested that sericin employees penetrating inflammatory cells at a low level similar to that of alginate and fibroin, but much less than the chitosan and it infact is able to recruit regeneration-promoting cells, such as vascular endothelial (progenitor) cells and it did not trigger any allergenic reaction, and demonstrated low and tolerable immunogenicity property. They confirmed that the sericin is a type of biosafe biomaterial for its potential biomedical applications. However, more detailed studies involving biosafety, pharmacodynamics, long-term exposure safety, immunogenicity and mechanism of action should be meticulously investigated using both in vitro and in vivo models before bring them to clinical trials. To date, there is no clinical trial studies involving sericin-based nanoformulations. Nevertheless, it is certain that the sericin-based nanoformulations will reach the clinics and contribute for future therapies of several diseases.

## Molecular mechanisms of SER-based nanoparticles

Although SER is widely used as a component of biomedical and cosmeceutical formulations, their molecular mechanisms remain unclear, especially regarding the SER differential properties capable to improve the nanoformulations therapeutic efficacy. Some reports discuss the SER hypothetical allergic activity; since immunogenicity is hardly exhibited in water-soluble silk SER [13, 100]. On the contrary, it has been described as easy healing of wounds, without inflammation effects induced by SER [101]. Moreover, SER enhanced the bioavailability of natural ingredients in foods, suppressed lipid peroxidation, and inhibited tyrosinase enzyme activity, which is advantageous for cosmeceutical applications [102]. Other reports revealed the prevention of cell death, the promotion of cell growth [103], and the attachment/proliferation of human skin fibroblasts [100].

The development of SER-nanoformulations must consider essential factors such as chemical structure conservation, the possibilities for designing different types of formulations (nanoparticles, micelles, films, hydrogels, etc.) according to the bioactive molecule to be incorporated or conjugated with SER, their biodegradability, the molecular mechanism responsible for in vitro*/*in vivo formulation therapeutic efficacy and also nanotoxicological aspects (biocompatibility and immunogenicity). Besides, the reactive oxygen species scavenging effects of SER based nanoparticles are attributed to SER amino acids primary structure, since the hydroxyl groups from serine and threonine act as chelating of copper, iron, and zinc metals; while other amino acids (alanine and glycine) present intracellular antioxidant effects [15, 17, 60]. In another report, antioxidant SER effects were demonstrated on mitochondrial structure preservation by regulating NADH-ubiquinone oxidoreductase, mitochondrial elongation factor Tu and prohibitin-2, intracellular proteins that regulate cell death events by apoptosis and autophagy. Besides, SER also regulates other enzymes activity such as aconitate hydratase and carnitine palmitoyltrasferase 2, acyl-CoA synthase, and beta-hydroxybutyrate dehydrogenase, modulating the lipids metabolism into hepatocytes [104]. All those factors exert a key outcome in successful nanoformulations to be considered for clinical trials as alternatives to traditional therapeutics.

## Other SER-formulations: hydrogels, films, and hybrid systems

The description of inherent SER biological activities associated with its easy functionalization created possibilities for designing many types of materials, including hydrogels, films, sponges, and dressing matrices, especially for skin tissue regeneration. SER-grafted conventionally used polymers (natural and synthetic polymers such as chitosan, alginate, gelatin, methacrylate) offered new aspects on the development of efficient materials for fibroblasts proliferation, dermal sealant, and human skin artificial equivalents. The most well-described matrices are hydrogels, designed for skin regeneration showed promising effects on injured skin favoring the appendages functional regeneration. Qi et al. [105], described the preparation of a UV photo-cross linked hydrogel formulation composed of SER-methacrylate, and evaluation in a skin injury model induced in mice. In fact, after hydrogel application, the skin regeneration index was improved and inflammation reduction was observed due to different molecular mechanisms including angiogenesis stimulation and enhanced the healing process by the vascular endothelial growth factor, TGF-β1, and β3 upregulation in addition to mesenchymal stem cells accumulation on damaged site. However, the use of synthetic polymers is discussed in the literature, and systems using SER-grafted natural polymers are proposed considering their non-immunogenicity and biocompatibility after the treatment of human dermal fibroblasts.

**Fig. 8:**
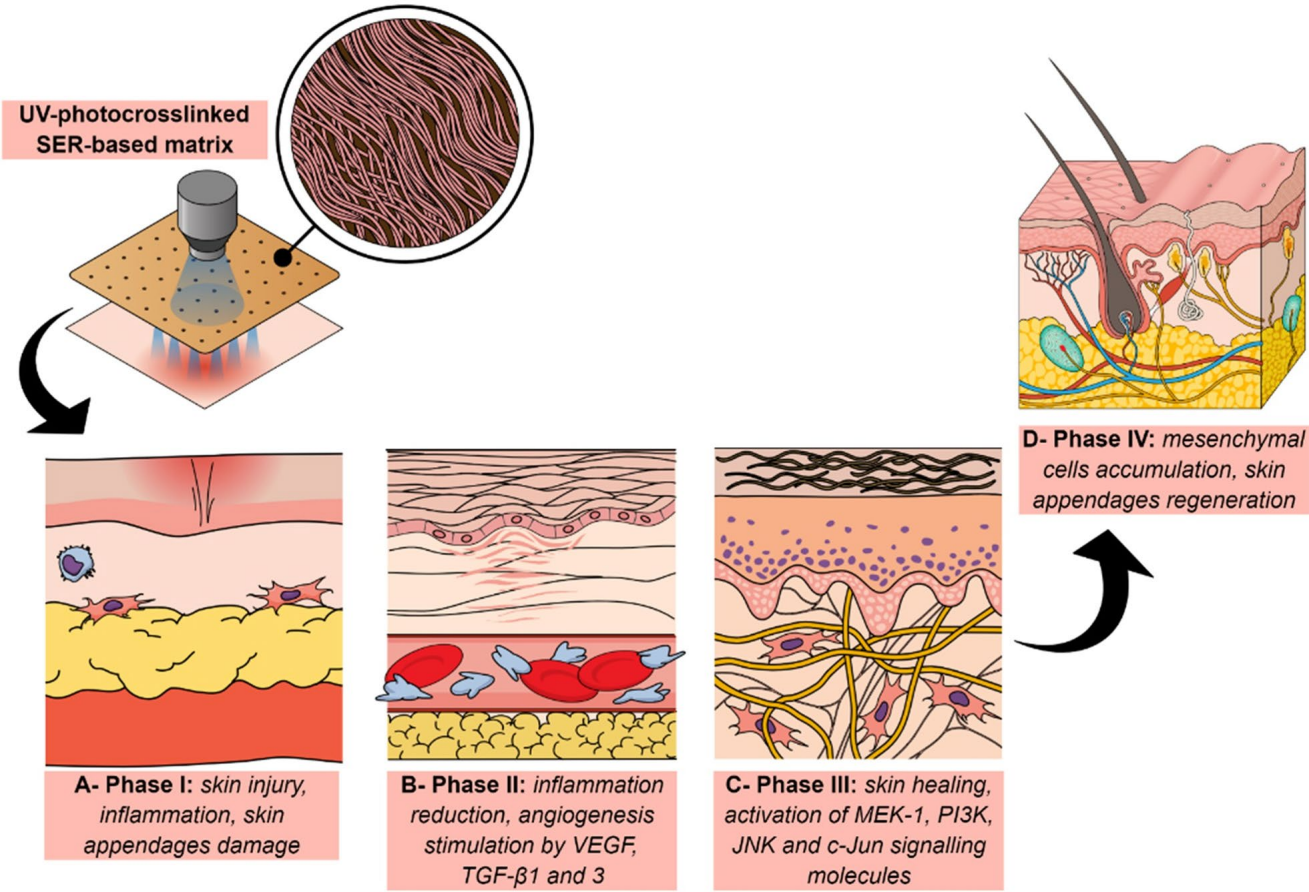
Representative application of UV-crosslinked SER-based formulation (hydrogels or films) and its molecular mechanisms on the skin regeneration process

As observed by Sapru et al. [106], SER-chitosan hydrogels induced no immunological response neither evoked cytokines production (TNF-α and IL1-β). The main advantages highlighted by authors are based on the reduced effective cost of the formulation and the biocompatibility provided using genipin, an aglycone compound, as a natural crosslinker. Subsequently, that formulation was evaluated in co-cultured human keratinocytes-dermal fibroblasts and as an implantable device by subcutaneous route demonstrating that the presence of SER induced skin cells adhesion and proliferation enhancing the collagen IV and metalloproteinases (types MMP2 and MMP9). All those integrated effects resulted in extracellular matrix and collagen network formation, responsible for skin regeneration [107]. Another effective natural blend for skin reconstruction applications was SER-gelatin as mechanically resistant and porous 3D-scaffolds or 2D-films, being cytocompatible matrices that promote skin fibroblasts proliferation without evoking cell cycle alterations[108]. Specific healing functions attributed to SER are related to an increase in collagen production, keratinocytes migration to the injured site, and release stimulation of signaling molecules (MEK1, PI3K, and JNK) able to activate c-Jun, important pathways for skin healing and keratinocytes migration [15, 17, 107]. As previously reported by Martinéz-Mora et al. [109], the treatment of epithelial breast cancer and mink lung epithelial cells with SER stimulated c-Jun, ERK 1/2 and JNK 1/2 kinases phosphorylation, initiating the cell migration intracellular events. In this sense, a recent innovation is devoted to the use of SER-alginate sponges for platelet growth factors delivery, promoting mesenchymal cells recruitment, tuning, and oxidative stress protection [110]. Figure 8 displays some representative mechanisms for SER-based systems applied to skin regeneration.

Several SER-matrices have been studied due to their adequate porosity, high permeability to water vapor, and oxygen, responsible for cell attachment and proliferative stimulation. All those features are proposed as essential requirements for blends biological effectivity. In addition to SER applications in the tissue repair sector, antimicrobial and cells targeting activities performed by multifunctional SER-biomaterials are also reported, characterizing the versatility of this protein. The association of two or more carriers into the same formulation results in a structurally complex system able to induce components (in general organic and inorganic) interactions and combine their biological activities. Thus, different SER-based hybrid systems have been pointed out as innovative strategies.

For example, SER-polyvinyl alcohol blends as film forms were studied as support for in situ production silver nanoparticles, which showed expressive antibacterial activity in *Escherichia coli* and *Staphylococcus aureus* cultures [5]. This method was called green biosynthesis due to the ability of phenolic hydroxyl groups from tyrosine residues on SER chemical structure to act as reductants of silver ions, dispersing and stabilizing silver nanoparticles and allowing the catalysis under natural light conditions [57]. Innovative, environmental-friendly, and low-cost SER-silver nanoparticle formulations represent recent efforts aiming at antimicrobial purposes, particularly against sexually transmitted pathogens, like HIV[42], as well as for obtaining synergistic antibacterial and burned skin regeneration[49]. Multifunctional SER-based formulations are complex structures produced by multi-steps synthesis reactions, such as SER-grafted fluorinated graphene oxide nanomedicines for cancel cells targeting by pH-responsive strategy via amide linkage hydrolysis into polyethyleneimine linker conjugated to SER molecule [89]. Another similar strategy was used by Qi et al. [111], for the production of a SER-methacryloyl-graphene oxide hydrogel. This innovative formulation was biocompatible and capable to induce the osteogenesis and cell differentiation processes via molecular mechanisms dependent on MAPK, TNF chemokines, and upregulation of mRNA levels for osteocalcin, collagen I, and transcription factor Runx2 after assessment in a rat calvarial bone repair model. The strategy of associating inorganic nanoparticles was also achieved during ophthalmic formulations development. Nagai et al. [48], assessed the therapeutic efficacy of a hybrid system containing nanoparticles composed of magnesium hydroxide and SER, for epithelial corneal healing lesions treatment, resulting in two complementary effects: the expansion of corneal intracellular space and corneal wound healing, evoked by magnesium hydroxide nanoparticles and SER, respectively. Then, SER-induced molecular mechanisms for epithelial corneal regeneration involves cellular essential events for cell adhesion and proliferation, such as phosphorylation of extracellular signal-regulated kinase – ERK.

It is already discussed that the nanoparticles size, surface charge, and coating, shape, and material, as well as their properties inherent to the cell type and environment, can influence nanoparticle uptake and their behavior and response in each cell type [112]. Cellular endocytosis occurs by two main mechanisms: phagocytosis, which is the mechanism used by dendritic cells and macrophages to engulf large particles and digest it while pinocytosis is responsible for the fluids internalization and small molecules inside small vesicles [113]. The last one can be organized into four mechanisms: macropinocytosis, clathrin-mediated endocytosis, caveolin-mediated endocytosis, and clathrin- and caveolin-independent endocytosis [114]. Several studies described the uptake mechanism of sericin-based nanoparticles. For example, Guo et al. [92], studied the uptake mechanism of sericin poly(γ-benzyl-l-glutamate) (PBLG) micelles in two cancerous cell lines, human mammary adenocarcinoma (MCF-7 ADR) and human hepatoma (HepG2). In both cell lines, the uptake mechanism was driven by clathrin-mediated endocytosis pathway. A similar endocytic pathway was observed for sericin nanoparticles coated with poly-l-lysine using a mouse fibroblast cell line (L929) [79]. In other studies, the cellular internalization of SER-folate nanoparticles was evaluated using human oral carcinoma cells (KB) and mouse fibroblast cell line (C2C12), which have a high and low level of folate receptors, respectively. Results pointed out that the nanoparticles uptake mainly driven by folate receptor since the nanoparticles uptake was very low in fibroblast cell lines and was drastically reduced in KB cells when free folate was added in the medium due to the competition to bind in the receptor [7]. To date, limited studies are evaluating the molecular mechanisms of SER-based nanoparticles. Most reports evaluated the increase of drug uptake due to nanoencapsulation but did not evaluate the mechanism driven the nanoformulation uptake.

## Conclusion and perspectives

Nanotechnological innovations have provided significant advances in fields of biomedicine and tissue engineering, especially considering the applications of a natural product for the development of new pharmaceutical formulations and biomaterials. Silk SER and its physicochemical properties have been described considering its appearance and extraction methods, especially because it is a byproduct obtained from silk production. Though some significant studies have been performed so far, still further detailed in vivo studies and clinical trials sericin based nanocomposites need to be carried out for their potential applications in cosmetics and pharmaceutical sectors. SER-based formulations are a great example of nanotechnological tools applied to the design of an economically viable, biocompatible, and biodegradable compound, as well as its use as nanomedicine. Despite some process limitations, SER isolation conditions have been studied and new solutions are proposed, including optimization on scale-up steps and production using transgenic silkworms, for example. All those innovations contribute to expanding SER applications in foods, cosmetics, and pharmaceutical fields. Additionally, SER has shown considerable vantages in comparison to other protein-derivatives, such as easy functionalization (forming blends with natural and synthetic polymers), FDA approval, biocompatibility, and inherent biological properties. As discussed herein, recent reports demonstrated SER relevant effects on cell adhesion, and proliferation, tissue repair (skin, bones, and joints), antioxidant, anti-inflammatory, antimicrobials, anti-tumoral, etc. Such mechanisms of action are dependent on protein structural features (molecular weight range, chemical structure conservation, and purity) and integrated intracellular sequential events since production and release of signaling molecules until up or downregulation of transcription factors. However, for nanomedicine applications, most studies are devoted to the development of new technologies associated with biocompatibility assays, early stages of the development, but for SER-based nanoformulations, the study of intracellular mechanisms of action is still a challenge. The vantages of those technologies are provided by SER ability to form supramolecular nanostructured new matrices which can exert differential effects on cell attachment (due to SER amino acids composition and scaffolds porosity, for example), shorten healing time compared to other treatments, being promising options for drug-delivery and pharmacotherapeutics regimens. In this sense, the knowledge about the SER-induced molecular mechanisms can contribute to enhance nanoformulations therapeutic activities and to promote synergistic effects with chemotherapeutic agents, for example, are essential. In this sense, future trends point out studies involving in vitro*/*in vivo relationships, immunogenicity, pharmacokinetics, and nanotoxicological aspects, providing information for possible clinical trials.

## Data Availability

All data related to the manuscript are available in the manuscript in the form of tables and figures.
